# The protective effects of repetitive transcranial magnetic stimulation with different high frequencies on motor functions in MPTP/probenecid induced Parkinsonism mouse models

**DOI:** 10.1002/brb3.3605

**Published:** 2024-07-02

**Authors:** Zhimai Lyu, Guodong Xiao, Dingyi Xie, Dandan Huang, Yanjun Chen, Chunmei Wu, Yanwei Lai, Zitan Song, Lijuan Huang, Hui Ming, Yichen Jiang, Jinwei Wang, Rixin Chen, Weifeng Luo

**Affiliations:** ^1^ Department of Neurology The First Affiliated Hospital of Gannan Medical University Ganzhou China; ^2^ Department of Neurology and Clinical Research Center of Neurological Disease The Second Affiliated Hospital of Soochow University Suzhou China; ^3^ The Ganzhou Key Laboratory of Noninvasive Neuromodulation Ganzhou China; ^4^ Department of Acupuncture and Moxibustion Affiliated Hospital of Jiangxi University of Chinese Medicine Nanchang China; ^5^ Department of Basic Medical Sciences Gannan Medical University Ganzhou China; ^6^ Department of International Exchange and Cooperation Jiangxi University of Chinese Medicine Nanchang China; ^7^ Department of Health Statistics, School of Public Health & Health Management Gannan Medical University Ganzhou China

**Keywords:** excitatory, high frequency, motor functions, Parkinson's disease, repeated transcranial magnetic stimulation

## Abstract

**Background:**

High‐frequency repeated transcranial magnetic stimulation (rTMS) stimulating the primary motor cortex (M1) is an alternative, adjunctive therapy for improving the motor symptoms of Parkinson's disease (PD). However, whether the high frequency of rTMS positively correlates to the improvement of motor symptoms of PD is still undecided. By controlling for other parameters, a disease animal model may be useful to compare the neuroprotective effects of different high frequencies of rTMS.

**Objective:**

The current exploratory study was designed to compare the protective effects of four common high frequencies of rTMS (5, 10, 15, and 20 Hz) and iTBS (a special form of high‐frequency rTMS) and explore the optimal high‐frequency rTMS on an animal PD model.

**Methods:**

Following high frequencies of rTMS application (twice a week for 5 weeks) in a MPTP/probenecid‐induced chronic PD model, the effects of the five protocols on motor behavior as well as dopaminergic neuron degeneration levels were identified. The underlying molecular mechanisms were further explored.

**Results:**

We found that all the high frequencies of rTMS had protective effects on the motor functions of PD models to varying degrees. Among them, the 10, 15, and 20 Hz rTMS interventions induced comparable preservation of motor function through the protection of nigrostriatal dopamine neurons. The enhancement of brain‐derived neurotrophic factor (BDNF), dopamine transporter (DAT), and vesicular monoamine transporter 2 (VMAT‐2) and the suppression of TNF‐α and IL‐1β in the nigrostriatum were involved in the process. The efficacy of iTBS was inferior to that of the above three protocols. The effect of 5 Hz rTMS protocol was weakest.

**Conclusions:**

Combined with the results of the present study and the possible side effects induced by rTMS, we concluded that 10 Hz might be the optimal stimulation frequency for preserving the motor functions of PD models using rTMS treatment.

## Graphical abstract

In order to figure out whether the high frequency of rTMS positively correlates to the improvement of motor symptoms of PD, we compared the protective effects of common high frequencies (5, 10, 15, and 20 Hz) of rTMS and iTBS (a special form of high‐frequency rTMS) on PD mice models. Taking into account the potential side effects caused by high frequency stimulation, we consider 10Hz to be the optimal stimulation frequency.

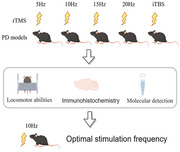

## 1 INTRODUCTION

Parkinson's disease (PD) is a slow, progressive neurodegenerative disease that affects close to 1% of the population over the age of 60, and males are more prevalent than females (Je et al., 2021; Vial et al., 2021). The number of patients increases year by year with age. The prevalence could be as high as 5% for people over the age of 85 (Qi et al., 2021). The major pathological hallmark of PD is the degeneration and necrosis of substantia nigra dopaminergic neurons. Motor symptoms such as bradykinesia, tremor, rigidity, and gait disturbance usually appear after the number of dopaminergic neurons drops by more than 50% (Lang & Obeso, 2004; Majali et al., 2021). The motor symptoms have become a serious medical issue and socioeconomic problem in many countries. A typical PD therapeutic approach is via pharmacological treatment such as dopamine supplementation or the administration of dopamine agonists (McDonald et al., 2018). However, with long‐term dopaminergic replacement therapy, adverse drug reactions or side effects have become common problems troubling Parkinson's patients (Fahn, 2015).

A number of nonpharmacological procedures have been suggested as alternative therapeutic strategies for PD, of which repetitive transcranial magnetic stimulation (rTMS) is one of the main prospective therapeutic methods (Goh et al., 2022). It is a noninvasive, painless, and safe neuromodulatory approach for modulating motor cortical excitability via plasticity‐like effects with the advantages of economy and convenience (Latorre et al., 2019). This technique has been considered to activate the dopaminergic pathways in the substantia nigra and striatum and promote the synthesis of brain‐derived neurotrophic factors, thereby improving the motor symptoms in PD (Chervyakov et al., 2015). Evidence‐based guidelines indicate that high‐frequency rTMS significantly reduced UPDRS‐III scores and improved the motor symptoms by stimulating the M1 target bilaterally in PD, and the level of evidence increased to Level B (Lefaucheur et al., 2020).

The frequencies of rTMS that are greater than 1 Hz are considered as high‐frequency rTMS. In the clinic, frequencies of rTMS applied commonly to the M1 area in the treatment of PD include 5, 10, 15, 20, and 25 Hz. Khedr et al. (2006) have conducted a study that compared the effects of 10 and 25 Hz rTMS over the M1 regions of PD patients for 3 months. The results showed that both protocols improved the motor symptoms of PD, and the effectiveness of 25 Hz rTMS was better than that of 10 Hz rTMS, suggesting that higher frequency rTMS might have better effect on the motor symptoms of PD. However, the effectiveness of 50 Hz rTMS over M1 has been demonstrated in a study on patients not receiving antiparkinsonian medications (Chung & Mak, 2016). The safety guidelines for the use of rTMS in clinical practice have pointed out that (Rossi et al., 2009) side effects induced by rTMS, such as headache and other discomforts, depend on multiple factors, of which the frequency of stimulation is one of the major factors. Moreover, the risk of epilepsy induced by rTMS rose with the increase in frequency. It seems that the obstacles faced in the clinical studies of rTMS increase when the frequency is above 20 Hz. The best frequency of rTMS, which is the most cost‐effective in improving the motor symptoms of PD, is still undecided. Further, the neuroprotective effects of different high frequencies of rTMS on PD‐related motor symptoms have not been compared in PD animal models.

**FIGURE 1:**
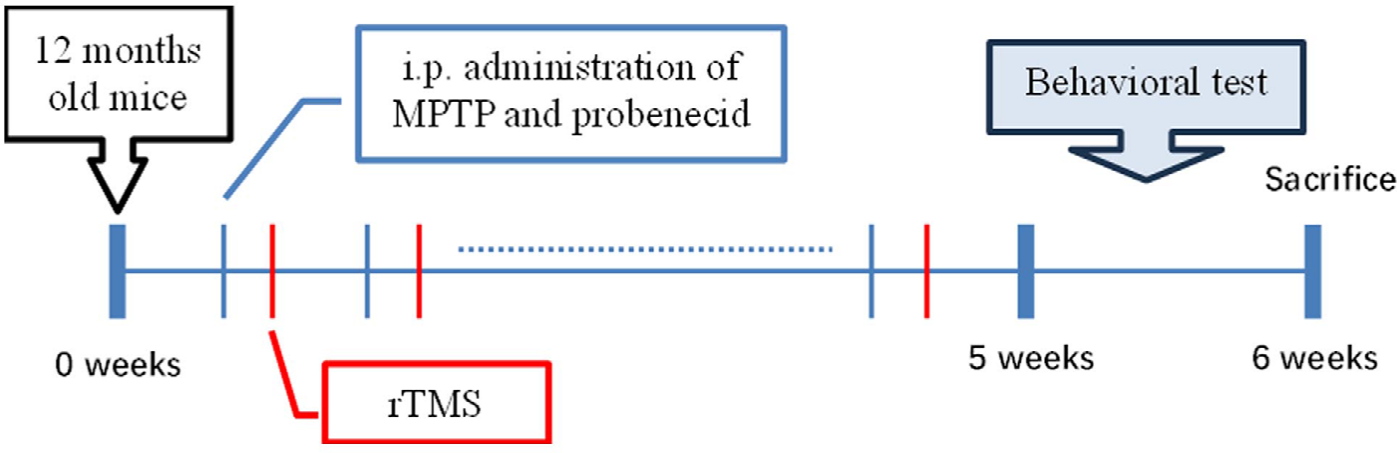
Experimental design. Parkinson's disease (PD) mouse model was induced by intraperitoneal (i.p.) administration of MPTP and probenecid (MPTP/p) twice a week for 5 weeks. The repetitive transcranial magnetic stimulation (rTMS) was delivered to the mouse on the day after the day of every administration of MPTP/p. The behavioral tests were carried out on the 6th week. After that, the mice were sacrificed and biochemical and molecular analyses were carried out.

The current study was, therefore, designed to compare the protective effects of four high‐frequency rTMS protocols (5, 10, 15, and 20 Hz) and iTBS (a special form of high‐frequency rTMS) in PD mice models induced by MPTP and probenecid. The effects of rTMS protocols were measured by behavioral assessments, including the rotarod test and the open field test. The neuroprotective effects on dopaminergic systems in the substantia nigra striatum and the related underlining mechanisms were also investigated (Figure 1).

## 2 MATERIALS AND METHODS

### 2.1 Animals

C57BL/6 aged male mice (*n* = 120), 25–30 g, approximately 12 months old, were kept in an appropriate animal room under a 12‐h light/dark cycle, with a room temperature of 22 ± 2°C, a humidity of 40%–70%, and free access to food and water. All the conditions of the animals in the experiment were in accordance with NIH guidelines and approved by the Animal Use and Care Committee of Jiangxi University of Traditional Chinese Medicine (No. 2021103001) for scientific purposes. All efforts were made to minimize the number of animals required in the present study.

### 2.2 Parkinsonian mouse model and grouping

The mice were rendered parkinsonian by the intraperitoneal (i.p.) administration of MPTP (20 mg/kg b.w. in saline; MCE) and probenecid (250 mg/kg in 0.03 mL DMSO; Sigma) for 5 weeks (twice a week, consisting of 10 injections in total). A control group received similar treatment with saline solution. To compare the protective effects of the four high frequencies of rTMS (5, 10, 15, and 20 Hz) and iTBS, the mice were randomly allocated to the following seven groups: control group, MPTP plus probenecid (MPTP/p) group, MPTP/p + 5 Hz rTMS group, MPTP/p + 10 Hz rTMS group, MPTP/p + 15 Hz rTMS group, MPTP/p + 20 Hz rTMS group, and MPTP/p + iTBS group (*n* = 14 for each group).

### 2.3 Repetitive transcranial magnetic stimulation

**FIGURE 2:**
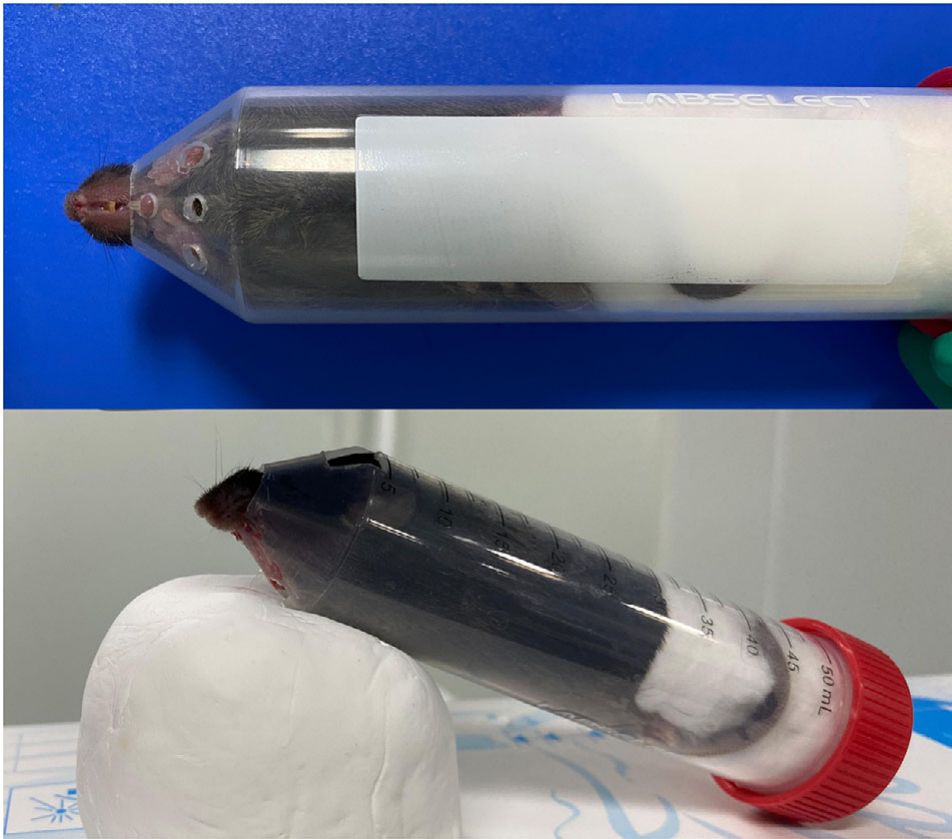
The image showed the restraint of awake mouse without anesthesia in a 50 mL‐centrifuge corning tube. The gaps left by the size differences in the mice within the tube can be adjusted using white and soft sponges as restraints.

The mice were immobilized with a 50‐mL centrifuge corning tube, which was suitable for the restraint of adult mice and commonly used in the laboratory (Zhang et al., 2019). The conical part at the bottom was suitable for accommodating the head of the mouse, while the circular tube was suitable for accommodating the body of the mouse. The difference in body size in the tube could be adjusted using a flexible sponge as a restraint (Figure 2). The rTMS was administered to awake mice by using a round coil (6.5 cm diameter) and a magnetic‐electric stimulator (CCY‐IA; Wuhan Yiruide Medical Equipment Co. Ltd.). The coil was placed 1.5 cm above the center of the exposed head and parallel to the parietal bone of the mice, which could ensure that the M1 areas of the mice received a field dose of approximately 1 T at least. The mice were exposed to four high frequencies of rTMS, respectively (5, 10, 15, and 20 Hz) and iTBS. For the conventional rTMS protocols, the parameters were as follows: 5/10/15/20 Hz, at 10‐s interval, a total of 1000 pulses for about 10 min, and 30% intensity of the machine output causing significant muscle twitching in the extremities of the mice. For the iTBS protocol, 20 trains of 50 Hz bursts (three pulses) repeated at 5 Hz were applied at 10‐s interval (600 stimuli). Stimulus strength was set at 30% of maximal device output. This stimulation was previously found to induce an electric field at the level of the subcortical white matter that is likely to stimulate the superficial cortical areas (Benali et al., 2011). To exclude the impact of sounds induced by rTMS on the mice, the mice in the control and MPTP/p groups were treated by the coil with the same protocol as the 10 Hz rTMS group but were placed 50 cm above the center of the exposed head. The rTMS was delivered to the mice on the day after the day of every administration of MPTP/p in order to assess the protective effects of rTMS on PD models. The i.p. administration of MPTP/p was twice a week for 5 weeks. Therefore, the rTMS treatments were performed for 10 sessions.

### 2.4 Open‐field tests

The locomotor abilities of mice were measured using open‐field tests. Mice were allowed to adapt to the environment for 30 min prior to testing. Then, they were placed individually in the center of a square box measuring 40 cm × 40 cm × 50 cm (JLBehv‐LAG‐4; Shanghai Yuyan Scientific Instrument Co. Ltd.) and kept there for 15 min. The movements and trajectories of the mice were monitored by video capture and an analysis system of animal behavior (Shanghai Jiliang Software Technology Co. Ltd.). The box was cleaned with 75% alcohol after each trial to remove body scents, which could be cues for other mice to move in subsequent evaluations.

### 2.5 Rotarod tests

Rotarod tests were applied to assess the motor deficiency of the mice. Briefly, mice were placed on a bar with a diameter of 3 cm and set 25 cm above the platform (YLS‐4C; Shanghai Xinruan Information Technology Co. Ltd.) at an accelerating speed (from 8 to 20 rpm in 300 s). The duration of their stay on the bar was recorded by a floor sensor, with a cutoff time of 300 s maintained throughout the experiment. The mice were trained for two consecutive days with two trials per day. Mice that did not reach a stable baseline performance were excluded from the experiment. After the training, the durations of staying on the bar on three trials were recorded formally on the third day and were averaged for the final statistical values. The bar was cleaned with 75% alcohol after each trial to remove body scents.

### 2.6 Immunohistochemistry

After behavioral testing, mice were anesthetized with an intraperitoneal injection of sodium pentobarbital (0.7%) at a dose of 50 mg/kg. Mice (*n* = 4 per group) were perfused intracardially with sterile saline and 4% paraformaldehyde (PFA) in 0.1 M PBS, and mice brains were rapidly removed and postfixed in 4% PFA for 24 h at 4°C and then cryoprotected with 30% sucrose until the brain sank to the bottom. The brains were sectioned serially into 30 µm‐thick sections through the midbrain. The free‐floating sections in 0.01 M PBS were exposed to 0.5% Triton X‐100 and 3% H2O2, blocked in 5% bovine serum albumin (BSA), and then incubated with rabbit polyclonal anti‐TH antibody (1:100) (25859‐1‐AP; Proteintech) and biotinylated goat anti‐rabbit antibody (1:200) (ZB‐2301; ZSGB‐BIO). Sections were developed with a DAB kit (CW0125M; CWBIO). Photomicrographs were captured by a Zeiss Axioplan 2 microscope. Then, the images of immunohistochemically stained slices were converted to grayscale and analyzed by ImageJ software (NIH) to measure TH immunoreactivities. The number of TH immunoreactive cells on each representative mesencephalic section was counted for the SN region. The cell counts were then averaged for each animal, and these averages were used to calculate a mean ± SEM for each treatment group, and the data were presented as a percentage of the control group.

### 2.7 Enzyme‐linked immunosorbent assay

The striatal areas of the mice from different groups were lysed and centrifuged at 4°C for 30 min to collect the supernatants (*n* = 6 per group). Following the manufacturer's instructions, quantitative determination of proinflammatory cytokines (TNF‐α and IL‐1β) in each sample was detected by the ELISA kits (MM‐0040M1, MM‐0132M1; Jiangsu Meimian Industrial Co. Ltd.). The optical density of each sample was determined by a microplate reader at 450 nm, and the levels of proinflammatory cytokines were calculated.

### 2.8 Western blot

The tissues from the substantia nigra and striatum were dissected from the brains and immediately frozen for protein extraction (*n* = 6 per group). The next procedure was performed the same as described previously (Chen et al., 2011). The membranes were probed with primary antibodies against TH (1:500, 25859‐1‐ap; Proteintech), BDNF (1:500, DF6387; Affinity), DAT (1:500, DF4529; Affinity), VMAT‐2 (1:500, DF7087; Affinity), and β‐actin (1:1000, TA‐09; ZSGB‐BIO). β‐Actin was used as a loading control. The proteins were detected using HRP‐conjugated anti‐rabbit secondary antibodies, visualized using chemiluminescence reagents provided with the ECL kit (WP20005; ThermoFisher Scientific), and exposed to film. The intensity of blots was quantified with densitometry (Quantity one; Bio‐Rad).

### 2.9 High‐performance liquid chromatography

The contents of dopamine (DA) and its metabolites, such as 3,4‐dihydroxyphenylacetic acid (DOPAC) and homovanillic acid (HVA) in striata (*n* = 4 per group), were analyzed by high‐performance liquid chromatography (HPLC) performed as previously described (Masoud et al., 2015). Briefly, samples of striata in each group were homogenized in 0.1 M perchloric acid/0.1% cysteine and centrifuged (18,000 r/min at 4°C for 20 min). The supernatants were filtered through a 0.22 µm membrane (Millipore) and analyzed using a TC‐C18 column (4.6 mm × 150 mm; 5 µm; Agilent) and a LC‐6A amperometric detector (Shimadzu). The standard curve and linear range were determined using DA, DOPAC, and HVA standards. DA levels were calculated from the standard curve equation and reported as ng/mg wet tissue.

### 2.10 Statistical analysis

All data are presented as the mean ± SEM and analyzed with GraphPad Prism 6.0. A one‐way analysis of variance was applied to analyze the data. For post hoc comparison, the Sidak post hoc test was used. A *p* value of <.05 was set as statistically significant.

## 3 RESULTS

### 3.1 Protective effects of high‐frequency rTMS on the motor functions in elderly chronic PD mice models

**FIGURE 3:**
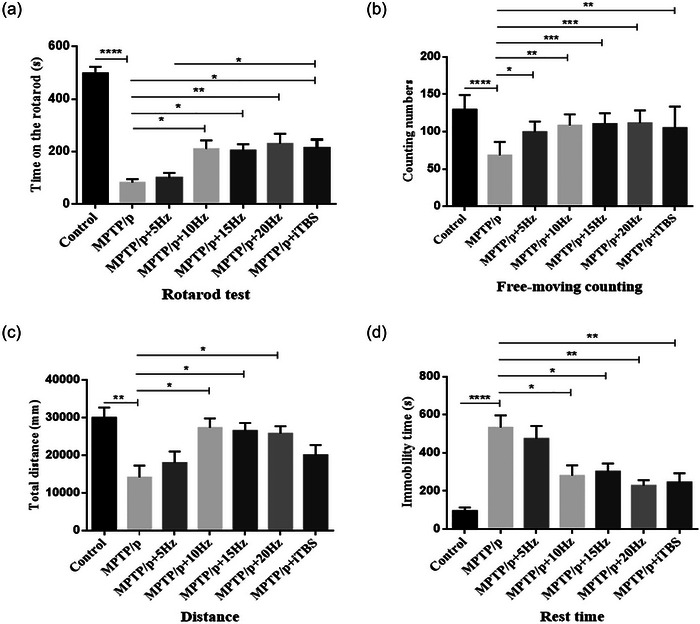
Effects of repetitive transcranial magnetic stimulation (rTMS) with different high frequencies on the motor functions in elderly chronic Parkinson's disease (PD) mice models. Rotarod tests (a) were performed to assess the motor functions. Free‐moving counting (b), total distance (c) and rest time (d) were also analyzed in open field tests to assess the motor functions. Values are expressed as the mean ± SEM. *p < .05, **p < .01, ***p < .001, ****p < .0001. A: *F*_(6,49) _= 24.84, B: *F*_(6,49) _= 8.002, C: *F*_(6,49) _= 4.942, and D: *F*_(6,49) _= 9.452.

In order to evaluate the protective effect of rTMS on the motor function of elderly chronic PD mice model, rotarod tests and open field tests were performed (Figure 3).

Time spent on the rotating platform of the rotarod test significantly decreased with MPTP/p compared to the controls (*p *< .0001). The free moving counting (*p *< .0001) and total distance (*p *= .0014) decreased and rest time increased (*p *< .0001) in the open field test with MPTP/p compared to the controls.

All the high‐frequency rTMS protocols applied in this experiment exhibited protective effects on the movement disorders of PD models in varying degrees. Among them, 5 Hz rTMS showed a unique attribute, that is, an increase in the free movement count in the open field test when compared to the MPTP/p group (*p* = .0226). The protocols of 10, 15, and 20 Hz rTMS were observed to increase the time spent on the rotating platform in the rotarod test (10 Hz *p* = .0272, 15 Hz *p* = .0399, 20 Hz *p* = .0065), raise the free movement count (10 Hz *p* = .0014, 15 Hz *p* = .0007, 20 Hz *p* = .0005) and total distance (10 Hz *p* = .0122, 15 Hz *p* = .0233, 20 Hz *p* = .0385), and reduce rest time (10 Hz *p* = .0101, 15 Hz *p* = .0243, 20 Hz *p* = .0010) in the open field tests compared to the MPTP/p group. In addition, the above three protocols showed no significant difference in improving motor‐like symptoms in the PD models. Noticeably, the iTBS protocol increased the time of the rotarod test (*p* = .0227) and the free movement count in the open field tests (*p* = .0041), and reduced the rest time in the open field tests (*p* = .0022) compared to the MPTP/p group. It showed a protective effect just behind the 10, 15, and 20 Hz rTMS protocols.

### 3.2 Protective effects of high‐frequency rTMS on dopaminergic nigrostriatal neurons

**FIGURE 4:**
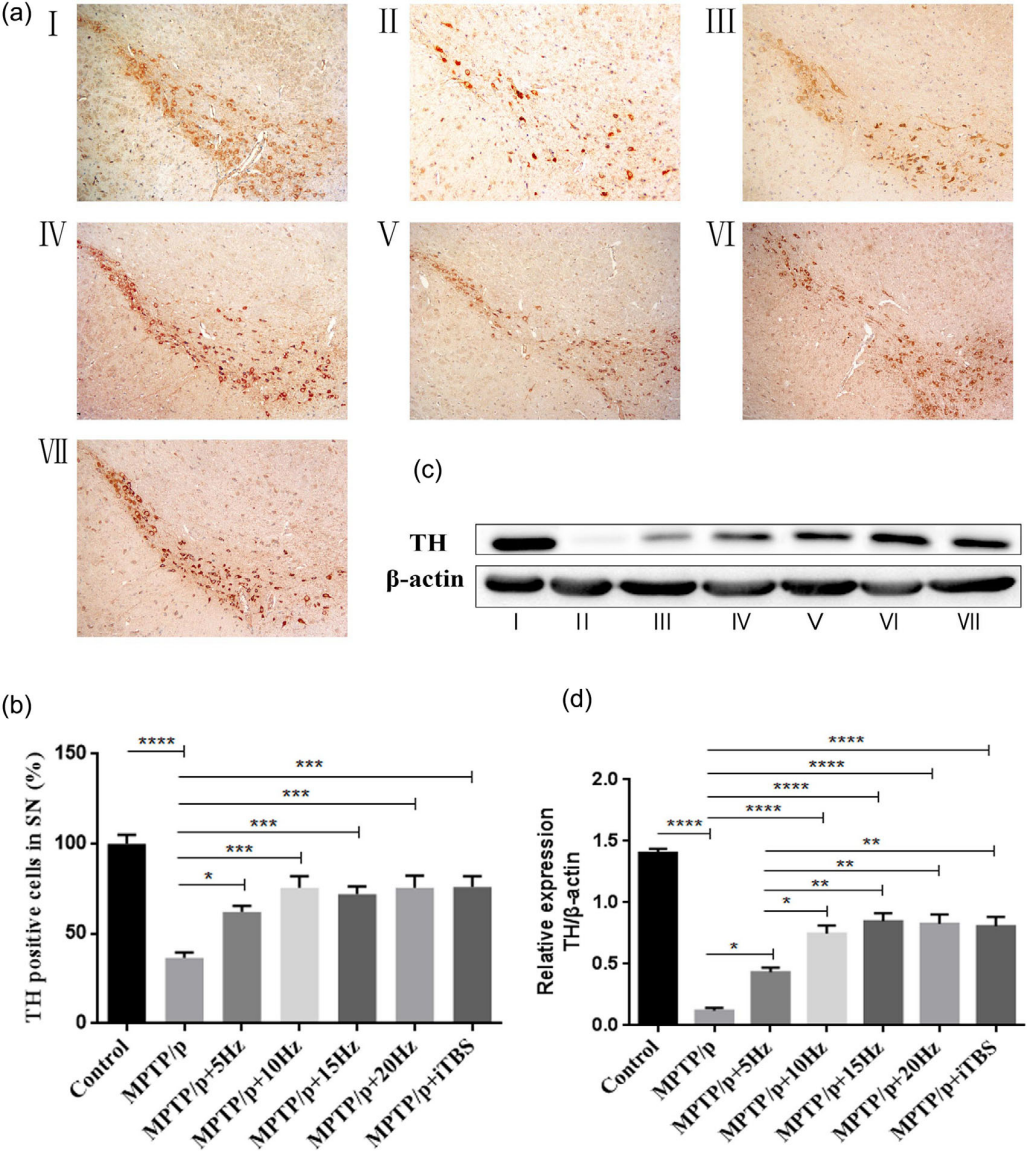
Effects of repetitive transcranial magnetic stimulation (rTMS) with different high frequencies on tyrosine hydroxylase (TH) expressions in elderly chronic Parkinson's disease (PD) mice models. Representative images of TH immunostaining (a) and western blot (c) in the substantia nigra in the groups were shown. The averaged data of TH‐positive neurons (b) and TH proteins (d) were displayed. Values are expressed as the mean ± SEM. *p < .05, **p < .01, ***p < .001, ****p < .0001. I: Control group; II: MPTP/p group; III: MPTP/p + 5 Hz group; IV: MPTP/p + 10 Hz group; V: MPTP/p + 15 Hz group; VI: MPTP/p + 20 Hz group; VII: MPTP/p + iTBS group. B: *F*_(6,35) _= 13.11, D: *F*_(6,49) _= 47.56.

**FIGURE 5:**
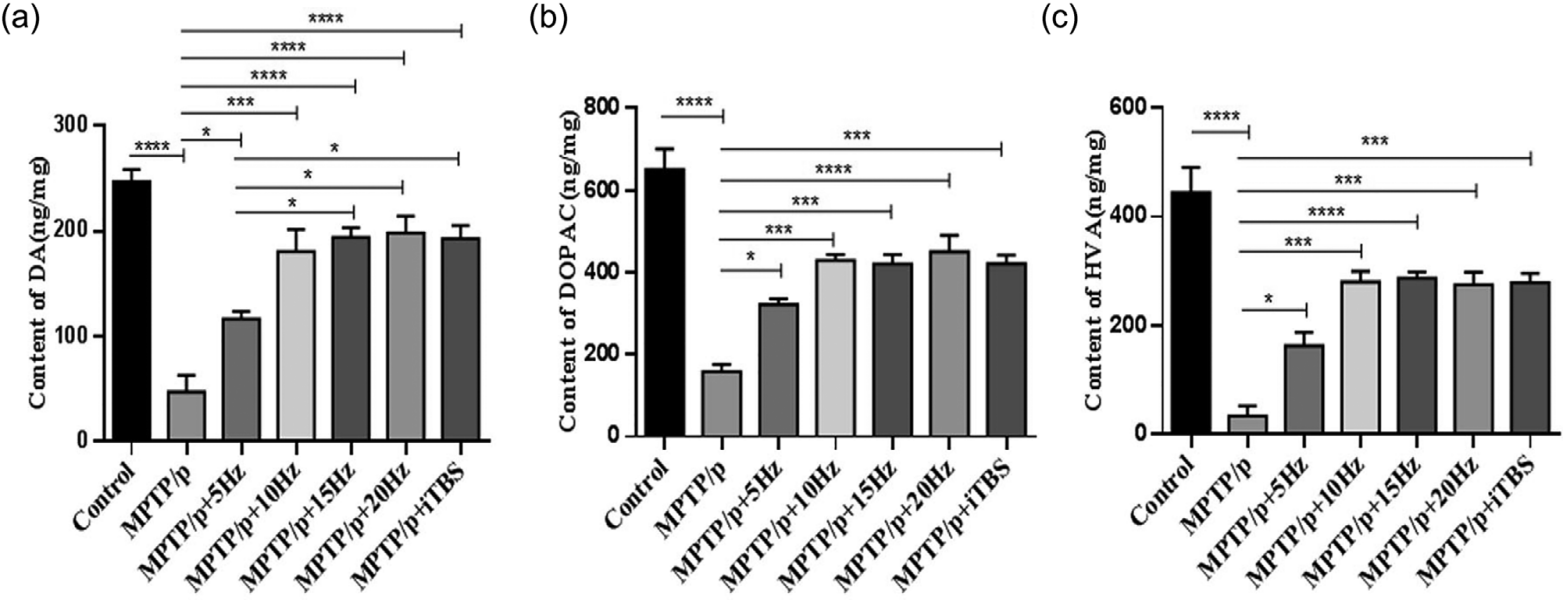
Effects of repetitive transcranial magnetic stimulation (rTMS) with different high frequencies on DA and its metabolites (DOPAC and HVA) in striata. The averaged data of DA (A), DOPAC (B) and HVA (C) in groups were displayed. Values are expressed as the mean ± SEM. *p < .05, ***p < .001, ****p < .0001. A: *F*_(6,14) _= 21.87, B: *F*_(6,14) _= 26.22, C: *F*_(6,14) _= 24.67.

The protective effect of high‐frequency rTMS on the DA neurons in the substantia nigra was evaluated by TH immunohistochemistry and western blot (Figure 4). Chronic MPTP/p administration led to a marked decrease in the number of TH positive neurons in the substantia nigra pars (SNc), compared to that of the controls (*p *< .0001). With the rTMS intervention, each of the protocols demonstrated significant enhancement in the number of TH positive neurons in the SNc, compared to that of the MPTP/p group (5 Hz *p* = .0327, 10 Hz *p* = .0002, 15 Hz *p* = .0006, 20 Hz *p* = .0002, iTBS *p* = .0001). There was no significant difference among the rTMS protocols. Western blot analysis demonstrated similar results when compared with the MPTP/p group (5 Hz *p* = .0240, 10 Hz *p *< .0001, 15 Hz *p *< .0001, 20 Hz *p *< .0001, iTBS *p *< .0001). In particular, the expression of TH protein in each of the three rTMS groups (10, 15, and 20 Hz) and iTBS was higher than that of the 5 Hz rTMS group (10 Hz *p* = .0344, 15 Hz *p* = .0034, 20 Hz *p* = .0051, and iTBS *p* = .0083). The striatal levels of DA and its metabolites (DOPAC and HVA) also reflected the function of dopaminergic nigrostriatal neurons. Results of HPLC showed that the levels of DA, DOPAC, and HVA decreased significantly in the MPTP/p group, compared to that of the controls (*p *< .0001). The stimulation induced by each rTMS protocol increased the levels of DA (5 Hz *p* = .0438, 10 Hz *p* = .0002, 15 Hz *p *< .0001, 20 Hz *p *< .0001, iTBS *p *< .0001), DOPAC (5 Hz *p* = .0169, 10 Hz *p* = .0002, 15 Hz *p* = .0003, 20 Hz *p *< .0001, iTBS *p* = .0002), and HVA (5 Hz *p* = .0367, 10 Hz *p* = .0001, 15 Hz *p *< .0001, 20 Hz *p* = .0002, iTBS *p* = .0001) significantly, compared to that of MPTP/p group. In particular, the levels of DA increased more in the 15 and 20 Hz rTMS and iTBS groups than in the 5 Hz rTMS group (15 Hz *p* = .0210, 20 Hz *p* = .0141, iTBS *p* = .0235) (Figure 5).

### 3.3 Protective effects of high‐frequency rTMS on DAT and VMAT‐2

**FIGURE 6:**
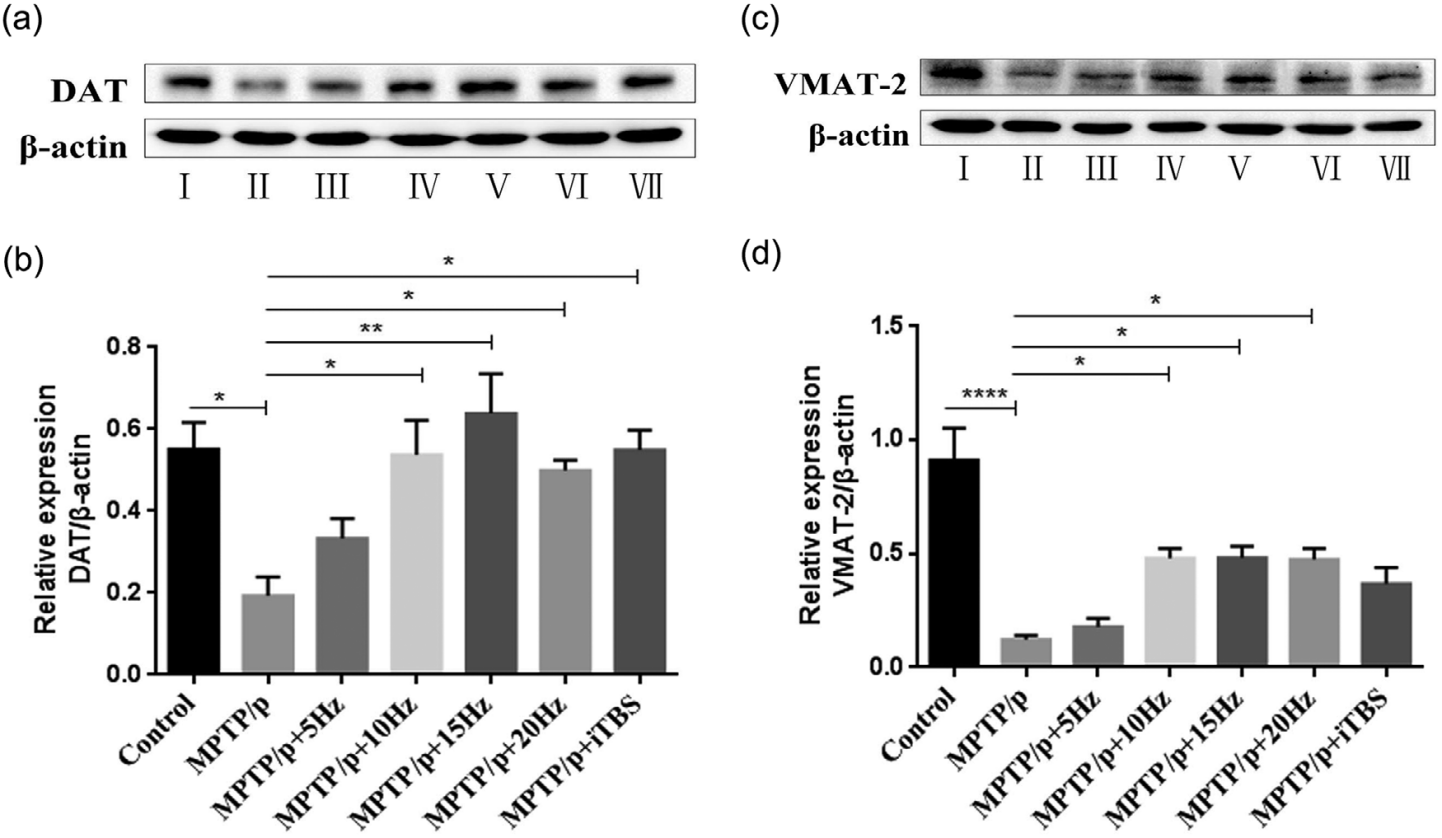
Effects of repetitive transcranial magnetic stimulation (rTMS) with different high frequencies on DA transporter proteins (DAT and VMAT‐2) in striata. Representative images of DAT (a) and VMAT‐2 (c) western blot in the striata were shown. The averaged data of DAT (b) and VMAT‐2 (d) proteins in groups were displayed. Values are expressed as the mean ± SEM. *p < .05, **p < .01, ****p < .0001. I: Control group; II: MPTP/p group; III: MPTP/p + 5 Hz group; IV: MPTP/p + 10 Hz group; V: MPTP/p + 15 Hz group; VI: MPTP/p + 20 Hz group; VII: MPTP/p + iTBS group. B: *F*_(6,14) _= 5.937, D: *F*_(6,14) _= 13.880.

DAT and VMAT‐2 are the two major DA transporter proteins in dopaminergic neurons (Klein et al., 2019; Stout et al., 2019). Measurements of the striatal levels of DAT (*p* = .0480) and VMAT‐2 (*p *< .0001) by western blot analysis revealed a significant decrease after MPTP/p administration. Except for the 5 Hz rTMS protocol, the other rTMS protocols increased the expression of DAT proteins significantly, compared to that of the MPTP/p group (5 Hz *p* = .6974, 10 Hz *p* = .0223, 15 Hz *p* = .0029, 20 Hz *p* = .0480, iTBS *p* = .0178). Similarly, the expression of VMAT‐2 proteins also increased in the 10, 15, and 20 Hz rTMS groups but not in the 5 Hz rTMS group, compared to the MPTP/p group (5 Hz *p* = .9970, 10 Hz *p* = .0333, 15 Hz *p* = .0327, 20 Hz *p* = .0373). However, the iTBS protocol showed no significant increase in the expression of VMAT‐2 when compared to the MPTP/p group (*p* = .2303) (Figure 6).

### 3.4 Suppression effects of high‐frequency rTMS on TNF‐α and IL‐1β levels

**FIGURE 7:**
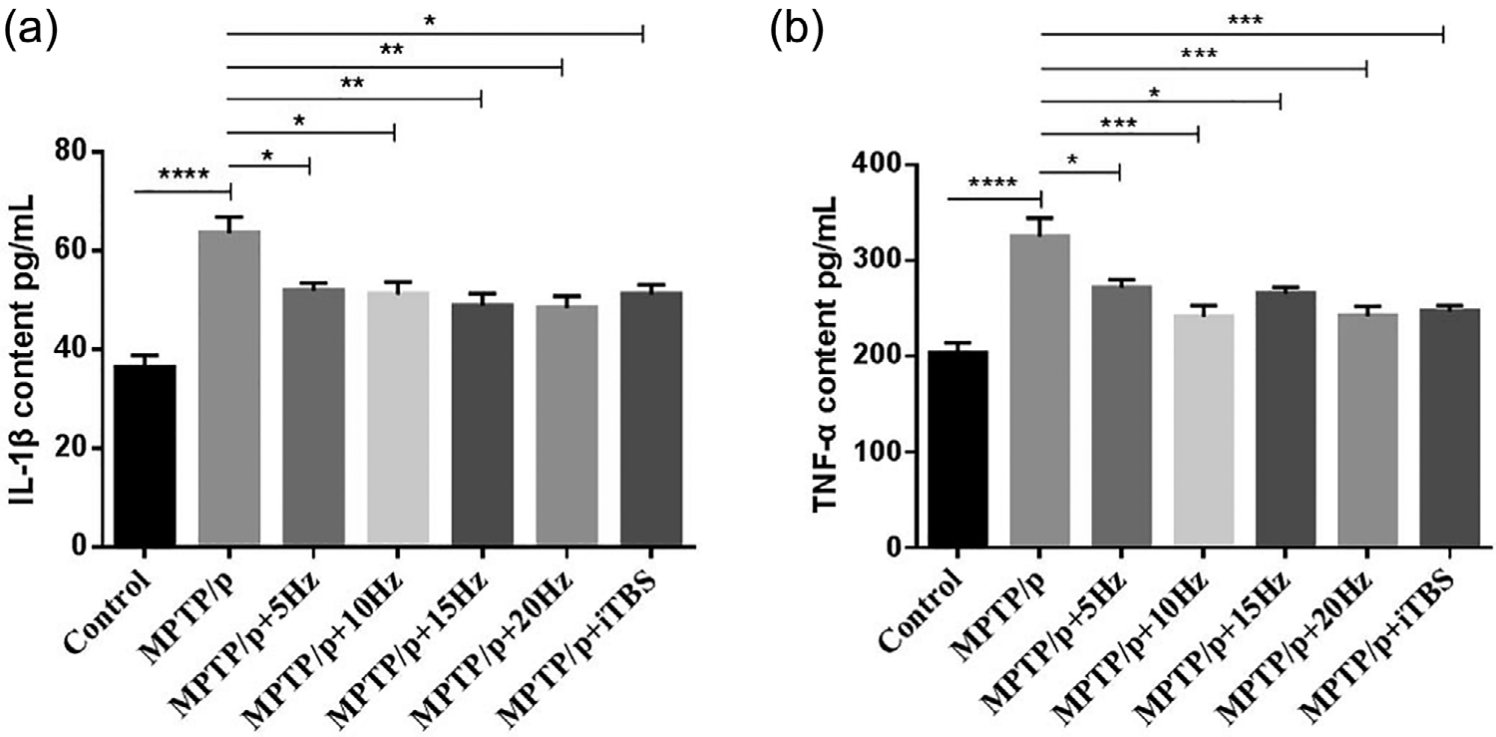
Effects of repetitive transcranial magnetic stimulation (rTMS) with different high frequencies on TNF‐α and IL‐1β in striata. The averaged data of TNF‐α (a) and IL‐1β (b) in groups were displayed. Values are expressed as the mean ± SEM. *p < .05, **p < .01, ***p < .001, ****p < .0001. A: *F*_(6,50) _= 8.222, B: *F*_(6,38) _= 9.987.

To evaluate the effect of high‐frequency rTMS on proinflammatory cytokines in PD mice, the striatal levels of TNF‐α and IL‐1β were measured using ELISA (Figure 7). The results revealed that MPTP/p administration significantly increased the levels of TNF‐α (*p *< .0001) and IL‐1β (*p *< .0001) when compared to control. With the rTMS intervention, the levels of TNF‐α (5 Hz *p* = .0250, 10 Hz *p* = .0003, 15 Hz *p* = .0219, 20 Hz *p* = .0004, iTBS *p* = .0008) and IL‐1β (5 Hz *p* = .0328, 10 Hz *p* = .0193, 15 Hz *p *= .0028, 20 Hz *p* = .0020, iTBS *p* = .0194) were significantly suppressed when compared to the MPTP/p group.

### 3.5 Protective effects of high‐frequency rTMS on BDNF in the substantia nigra striatum

**FIGURE 8:**
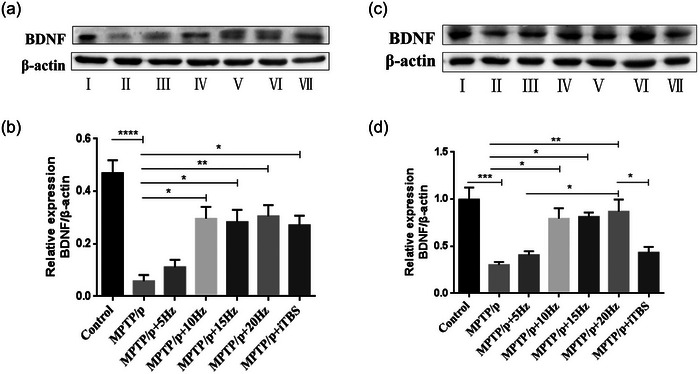
Effects of repetitive transcranial magnetic stimulation (rTMS) with different high frequencies on BDNF protein in the substantia nigra and striatum. Representative BDNF western blot images in the substantia nigra (a) and striata (c) in the groups were shown. The averaged data of BDNF proteins in the substantia nigra (b) and striata (d) were displayed. Values are expressed as the mean ± SEM. *p < .05, **p < .01, ***p < .001, ****p < .0001. I: Control group; II: MPTP/p group; III: MPTP/p + 5 Hz group; IV: MPTP/p + 10 Hz group; V: MPTP/p + 15 Hz group; VI: MPTP/p + 20 Hz group; VII: MPTP/p + iTBS group. B: *F*_(6,14) _= 11.760, D: *F*_(6,14) _= 9.717.

BDNF is highly expressed in substantia nigra dopaminergic neurons, and it plays an important role in the maintenance and differentiation of dopaminergic neurons (Zuccato & Cattaneo, 2009). After MPTP/p administration, BDNF decreased significantly in the substantia nigra (*p *< .0001) and striatum (*p* = .0009) when compared to the controls. With the rTMS intervention, the expression of BDNF in substantia nigra increased in the 10, 15, and 20 Hz rTMS and iTBS groups except in the 5 Hz rTMS group, compared to the MPTP/p group (5 Hz *p* = .9571, 10 Hz *p* = .0112, 15 Hz *p* = .0171, 20 Hz *p* = .0081, iTBS *p* = .0251). Similarly, the expression of BDNF in the striatum also increased in the 10, 15, and 20 Hz rTMS groups, compared to the MPTP/p group (10 Hz *p* = .0176, 15 Hz *p* = .0131, 20 Hz *p* = .0058). However, both of the 5 Hz rTMS (5 Hz *p* = .9755) and iTBS (iTBS *p* = .9253) protocols had no effect on enhancing the expression of BDNF in the striatum (Figure 8).

## 4 DISCUSSION

Evidence‐based guidelines have indicated that high‐frequency rTMS significantly reduced UPDRS‐III scores and improved the motor symptoms by stimulating M1 targets bilaterally in PD patients (Lefaucheur et al., 2020). However, a frequency greater than 1 Hz is considered to be high‐frequency. The best high‐frequency rTMS for improving the motor symptoms of PD is still undecided. This gap in knowledge is probably due to variability across therapeutic protocols, medication conditions, clinical heterogeneity, and disease severity (Yang et al., 2018). An animal model could be helpful for controlling factors and may provide more information for clarifying the best high‐frequency and underlying mechanism of applying rTMS to PD patients. In the present study, we compared the protective effects of four common high‐frequency rTMS protocols (5, 10, 15, and 20 Hz) and iTBS on the motor function of PD mice models, so as to explore the best high‐frequency rTMS for PD treatment.

Among the different PD animal models, the systemic administration of MPTP is the most widely used protocol to induce nigrostriatal degeneration and mimic clinical Parkinsonism, especially the chronic administration of MPTP protocol (Muñoz‐Manchado et al., 2016). Some studies added probenecid to improve MPTP neurotoxicity by inhibiting central MPP+ clearance and/or renal MPTP excretion. The subsequent result was to increase MPP+ concentration available to the brain cells. The MPTP/probenecid (MPTP/p) protocol has proven to exacerbate neurotoxicity in mice, resulting in a more pronounced and prolonged DA depletion compared to that with an MPTP‐only protocol (Wada et al., 2021). Ageing is also a major risk factor for PD and sharing cellular mechanisms with the degeneration of dopaminergic nigral neurons (Collier et al., 2011). For the above reasons, this study used the chronic intraperitoneal administration of MPTP/p protocol to produce the PD models in aged C57BL/6 mice (12 months old), so as to mimic the features of PD as much as possible. In fact, this experiment demonstrated good results in rendering PD features.

Frequencies of more than 20 Hz, such as 25 Hz or higher frequencies, are prone to induce headache, nausea, and scalp discomfort in rTMS treatment. For this reason, we did not use 25 Hz or higher frequencies of rTMS in this study. The high frequencies of rTMS used in this study were commonly utilized in clinic rTMS treatments. The iTBS, an intermittent theta burst protocol based on the endogenous neural theta rhythm, induced facilitation or LTP‐like plasticity that was similar to the physiological effect of high‐frequency rTMS (Ghiglieri et al., 2012). It was also used in this study as a special high‐frequency protocol. Generally, stimulus intensity required for iTBS is lower compared to the rTMS protocols (Huang et al., 2005). In line with the parameters of other rTMS protocols as much as possible, however, the same stimulus strength (30% of maximal device output) was set in iTBS protocol.

In this experiment, a circular stimulation coil was placed 15 mm above the center of the mouse's head, generating the strongest magnetic field covering the bilateral M1 areas of the mouse, which effectively induced bilateral forelimb movements. Although some frontal and parietal lobes are also affected by the magnetic field, when viewed along the cortical surface of the mouse, the intensity of the magnetic field is lower anteriorly and posteriorly while higher in the middle; hence, other areas receive weaker magnetic field strength compared to the M1 area, and the main effect area can still be considered as the bilateral M1 area. Additionally, in our clinical work, we found that the stimulation effect of the circular coil is better than that of the figure‐eight coil. The reason may be that when the target area is subcortical, although rTMS stimulates the cerebral cortex, it needs to affect the target area through neural circuits; theoretically, the stimulation effect of reaching the target area through multiple neural circuits should be better than a single neural circuit, and the circular coil has a wider range of stimulation compared to the figure‐eight coil, affecting more neural circuits. The stimulation area in this experiment mainly targets the M1 area, with other areas playing a secondary role. Although such stimulation patterns lack precise specificity, this study has attempted to be relatively precise for observing the neuroprotective effects of rTMS on MPTP‐induced PD under limited experimental conditions. Moreover, the substantia nigra region in mice is very small, and even with the smallest animal stimulation coil available, it is difficult to ensure precise stimulation of the motor cortex‐nigra pathway. In fact, although the data published in this experiment focus on the improvement of motor functions and PD‐related molecular changes associated with rTMS whole‐brain effects, other non‐motor symptoms, such as tail suspension test and forced swimming test, also showed significant improvement, but are not the focus of this paper. Therefore, as long as it does not affect the experimental purpose, nonspecific whole‐brain effects also have their advantages. After all, achieving specificity in transcranial magnetic stimulation for the mouse brain is inherently challenging.

After the rTMS interventions, we found that all the rTMS protocols mitigated the motor‐like symptoms of PD models to varying degrees. Among them, the effects of the 10, 15, and 20 Hz rTMS protocols were obvious, followed by the effect of the iTBS protocol. The effect of the 5 Hz rTMS protocol was the weakest. There was no significant difference among the 10, 15, and 20 Hz rTMS protocols. In the clinic, the higher the frequency of stimulation, the more likely it is to cause discomfort, and the possibility of inducing epilepsy increases. From this perspective, it seems that 10 Hz rTMS might be the best high‐frequency rTMS in preserving motor functions of PD animal models.

The expressions of TH, DA, and their metabolites in the nigrostriatum were detected in all groups. The results revealed that all the rTMS interventions used in this experiment demonstrated significant performance enhancement in the expressions of TH, DA, and their metabolites in the nigrostriatum, which mainly contributed to preserving the motor functions of PD models. Particularly, the effect of each protocol (10, 15, and 20 Hz rTMS and iTBS) was much better than that of the 5 Hz rTMS, and there was no significant difference among the 10, 15, and 20 Hz rTMS and iTBS protocols. This indicated that the effect of the 10 Hz rTMS protocol on protecting the dopaminergic neurons was statistically no different from that of the others.

BDNF is a potent inhibitor of apoptosis and neurotoxin‐induced degeneration of dopaminergic neurons. Due to its crucial effects on the survival and morphology of dopaminergic neurons, a loss of BDNF may contribute to the death of these neurons and the subsequent pathogenesis of PD (Zhao et al., 2017). In this study, the 10, 15, and 20 Hz rTMS protocols improved the expression of BDNF protein in the nigrostriatum. There was no difference among those three protocols. We speculated that the dopaminergic neuroprotective effects of the above three protocols were highly related to the enhancement of BDNF expression. DA can be both a neurotransmitter and a neurotoxin (Segura‐Aguilar et al., 2014). It can exert toxicities by accelerating redox processes, forming protein adducts in some cellular environments, and altering synuclein aggregation (Masato et al., 2019; Mor et al., 2017; Yu et al., 2015). When pumped timely into synaptic vesicles by the synaptic VMAT2 and into the intracellular/extravesicular cytosolic compartment by the plasma membrane DAT, DA is unlikely to exert such toxic activities (Uhl, 2019). In this study, the expressions of DAT and VMAT2 in the striatum were enhanced, respectively, in the 10, 15, and 20 Hz rTMS groups, compared to the MPTP/p group. Enhancing the expressions of DAT and VMAT2 was attributed to dopamine neuronal survival. Regarding the effect of the iTBS protocol on BDNF, DAT, and VMAT‐2, it simply increased the expression of BDNF in the substantia nigra and DAT in the striatum. This may be the reason why the motor behavioral preservation of PD mice with the intervention of the iTBS protocol was inferior to that of the other three protocols (10, 15, and 20 Hz rTMS) in this experiment. The 5 Hz rTMS protocol had no impact on expression of BDNF in the nigrostriatum, as well as DAT and VMAT‐2 in the striatum. However, all the rTMS protocols reduced the expressions of TNF‐α and IL‐1β in the striatum, and there was no difference among those protocols in this study. Central nervous system inflammation is considered to be involved in the neurodegenerative process of PD. BDNF participates in phagocytosis to remove damaged neurons and foreign matters, and participates in immune monitoring by secreting anti‐inflammatory or pro‐inflammatory cytokines. The pro‐inflammatory cytokines, such as TNF‐α and IL‐1β, have potential neurotoxicity and aggravate the pathology of PD (Cinar et al., 2022). In this study, we inferred that the dopaminergic neuroprotective effects of 5 Hz rTMS were probably mainly ascribed to its anti‐neuroinflammatory effect.

To sum up, combined with the behavioral results and molecular biological detections, this study indicated that 10, 15, and 20 Hz rTMS interventions induced comparable preservation of motor functions through the protection of the nigrostriatal dopamine neurons. The neuroprotective effects on dopaminergic cells might be related to the enhancement of BDNF, DAT, and VMAT‐2 and the suppression of TNF‐α and IL‐1β in the nigrostriatum. Although the 5 Hz rTMS and the iTBS interventions also protected the nigrostriatal dopamine neurons and preserved the motor functions in PD models, their efficacy was inferior to that of the others. In view of the probability of discomfort and epilepsy induced by high‐frequency rTMS, we considered that 10 Hz rTMS might be the optimal high‐frequency rTMS for the preservation of motor function in PD animal models.

## 5 CONCLUSION

This exploratory study compared the protective effect of four common high frequencies of rTMS (5, 10, 15, and 20 Hz) and iTBS protocols on the motor function of PD mice models and explored the underlying mechanisms of the neuroprotective effects. Combined with the behavioral results and possible side effects induced by rTMS, we concluded that 10 Hz might be the optimal frequency for preserving motor function in PD animal models with rTMS treatments. This protective effect was related to increasing the expression of TH, BDNF, DAT, VMAT‐2, DA, and their metabolites and reducing the expression of TNF‐α and IL‐1β in the nigrostriatum. Future clinical studies are needed to verify the advantage of the curative effect for 10 Hz rTMS in PD patients.

## AUTHOR CONTRIBUTIONS

**Zhimai Lyu**: Conceptualization; methodology; investigation; formal analysis; funding acquisition; writing—original draft; data curation. **Guodong Xiao**: Conceptualization; validation; investigation; writing—original draft; formal analysis; data curation. **Dingyi Xie**: Investigation; writing—original draft; conceptualization. **Dandan Huang**: Investigation; methodology; formal analysis. **Yanjun Chen**: Investigation; methodology; writing—review and editing. **Chunmei Wu**: Data curation; formal analysis. **Yanwei Lai**: Data curation; formal analysis. **Zitan Song**: Investigation; data curation. **Lijuan Huang**: Investigation; data curation. **Hui Ming**: Investigation; data curation. **Yichen Jiang**: Investigation. **Jinwei Wang**: Investigation. **Rixin Chen**: Investigation; resources; supervision; funding acquisition; writing—review and editing. **Weifeng Luo**: Conceptualization; methodology; resources; writing—review and editing; project administration; supervision.

## CONFLICT OF INTEREST STATEMENT

The authors declare no conflicts of interest.

### PEER REVIEW

The peer review history for this article is available at https://publons.com/publon/10.1002/brb3.3605.

## Data Availability

The data that support the findings of this study are available from the corresponding author upon reasonable request.
